# Effect of Pilocarpine Mouthwash on Salivary Flow Rate in Patients with Xerostomia: A Systematic Review and Meta-Analysis

**DOI:** 10.30476/dentjods.2022.94335.1778

**Published:** 2023-03

**Authors:** Katayoun Katebi, Shirin Hassanpour, Hosein Eslami, Fatemeh Salehnia, Hossein Hosseinifard

**Affiliations:** 1 Dept. of Oral and Maxillofacial Medicine, Faculty of Dentistry, Tabriz University of Medical Sciences, Tabriz, Iran; 2 Postgraduate Student, Dept. of Pediatric Dentistry, Faculty of Dentistry, Tabriz University of Medical Sciences, Tabriz, Iran; 3 Research Center for Evidence Based Medicine (RCEB), Tabriz University of Medical Sciences, Tabriz, Iran

**Keywords:** Pilocarpine, Xerostomia, Saliva, Mouthwashes

## Abstract

**Statement of the Problem:**

Xerostomia is a subjective sensation of dry mouth considered as a complex state affecting multiple oral functions. Pilocarpine may be a useful medication for the treatment of xerostomia, but its side effects limit its use under certain conditions. Recent studies have focused on the pilocarpine mouthwash as an alternative.

**Purpose:**

We have undertaken this study to review the latest available scientific evidence systematically, concerning the effects of pilocarpine mouthwash on salivary flow rate in patients with xerostomia.

**Materials and Method:**

An electronic search for randomized controlled trials published in English until September 2021 related to pilocarpine mouthwash and salivary flow rate in patients with dry mouth was performed in PubMed/Medline, Web of Science, Google Scholar, Embase, and Scopus. A random-effects meta-analysis was conducted to evaluate the relationship between the groups.

**Results:**

Two papers with 86 patients were selected for the final review based on strict eligibility criteria. According to the results of the meta-analysis, the mean visual analogue scale in the patient treated with pilocarpine mouthwash was 0.88 unit lower than that of the control group in the fourth week follow; however, it was not statistically significant (pooled mean difference=-0.88, 95% CI = (-2.72; 0.95), *p*= 0.34).

**Conclusion:**

It seems that the use of pilocarpine mouthwash can increase the salivary flow rates; however, no optimal dose and application regimen can currently be suggested due to the high heterogeneity of the data. Regarding the relief of the symptoms using pilocarpine mouthwash, the existing evidence does not support its effectiveness.

## Introduction

Xerostomia is a subjective sensation of dry mouth considered as a complex state affecting multiple oral functions [ 1 - 2 ]. This condition can be related to the hypofunction of the salivary gland [ 3 - 4 ]. Since saliva plays an essential role in maintaining oral health, long-term xerostomia affects the quality of life, leading to difficulty in swallowing, susceptibility to infections, and several oral problems, such as tooth decay, periodontal diseases, and candidiasis [ 5 - 10 ]. The etiology of xerostomia appears to be multifactorial, where local and systemic factors may be involved [ 11 - 12 ], among which the most common cause involves the adverse effects of various prescription and over-the-counter medications [ 13 ]. Other common causes include Sjögren’s syndrome, head and neck radiotherapy, dehydration, smoking, and the inflammation or the infection of the salivary glands [ 14 - 18 ]. Several treatment options have been assessed for xerostomia including, humidifiers, artificial saliva, chewing gum, sugar free lozenges, ginger capsule and so on [ 19 - 22 ], among which pilocarpine has attracted a lot of attention. This cholinergic agonist binds to muscarinic receptors, promotes the secretion of salivary glands, and its effectiveness on radiotherapy-induced xerostomia and Sjögren’s syndrome has been shown in several studies [ 23 - 28 ]. However, because of its non-selective action, the main reported negative effects include excessive sweating, nausea, vomiting, diarrhea, and headache [ 29 - 31 ]. Furthermore, pilocarpine should be prescribed with vigilance in patients with asthma, chronic obstructive pulmonary disease, and cardiovascular disease, and its use is contraindicated in patients with acute asthma attacks, narrow-angle glaucoma, iritis, and in elderly individuals who may be exposed to polypharmacy [ 32 - 34 ]. As a result, while pilocarpine may be a useful medication for the treatment of xerostomia, its side effects limit its use under certain conditions.

 In this respect, recent studies have focused on the topical application of pilocarpine as an alternative with the advantage of minimizing its adverse effects [ 35 - 38 ]. Kim *et al*. [ 37 ] evaluated the effectiveness of pilocarpine mouthwash in dry mouth. Sixty volunteers were treated with pilocarpine or placebo. As a mouthwash, pilocarpine increased saliva secretion from minor salivary glands more than the placebo. Tanigawa *et al*. [ 38 ] assessed the efficacy of 0.01% pilocarpine as mouthwash in 40 elderly individuals who were divided into pilocarpine and placebo groups randomly. Their results showed that pilocarpine mouthwash reduced the symptoms of dry mouth and increased the salivary flow with minimal side effects.

Randomized clinical trials focused on assessing the effectiveness of topical pilocarpine for the management of xerostomia have used different regimens of medication and produced different results. We have undertaken this study to review the latest existing scientific evidence regarding the effects of pilocarpine mouthwash on patients with xerostomia. 

## Materials and Method

This study has been approved by the Regional Ethics Committee (IR.TBZMED.REC.1400.203). The study protocol is registered in PROSPERO with registration number of CRD42021282073.

In this systematic review, the principal question was formulated on the basis of the “PICO” (population, intervention, comparison, and outcome) approach, where “P” was patients with xerostomia, “I” was pilocarpine mouthwash, “C” was placebo, and “O” was the improvement of dry mouth symptoms and increased salivary volume. The purpose of this study was to determine if pilocarpine as mouthwash could improve the symptoms of xerostomia and increase the salivary volume in patients with xerostomia compared to the placebo. 

### Inclusion Criteria

The inclusion criteria were defined as:

1. Double-blind randomized controlled trials and cohort studies 

2. Only studies focusing on patients with xerostomia 

3. Only studies using pilocarpine as mouthwash 

4. Only English papers 

5. Papers published until October 2021 

### Databases and Search Strategy

The article selection process was performed in four steps according to the PRISMA flow diagram [ 39 ]. A librarian (F.S) conducted the electronic search.

PubMed/Medline, Web of Science, Google Scholar, Embase, and Scopus databases were searched. The keywords were selected from Medical Subject Heading (MESH) terms. The search keywords were “xerostomia”, “dry mouth”, “oral dryness”, “mouth dryness”, “saliva”, “spittle”, “pilocarpine”, “mouthwash”, “mouth rinse”, “eye drop”, “topical”, “placebo”, “normal saline”, “physiological saline”, “saline solution”, “saline solution, hypertonic”, “sodium chloride”, “saliva substitute”, and “artificial saliva”, “visual analog scale”.

Every possible combination of free and MESH (Medical Subject Heading) terms with “OR” and “AND” operators was considered for finding the data. The exact search terms are provided in supplement file. The research team made an effort to communicate with the corresponding authors for supplementary information if necessary. To identify more research studies, the reference lists of the selected studies were searched as well. 

The EndNote Basic software was used to manage the references, and duplicate references were identified and removed.

### Study Selection

Two independent reviewers (K.K and S.H) scanned the titles and abstracts of the articles independently. Afterwards, the full texts of the selected articles were reviewed. In the case of a disagreement between two reviewers, a third reviewer (H.E) was consulted. Finally, the full-text evaluation of the included articles was performed using a pre-designed data extraction sheet. 

### Assessment of the Risk of Bias

The revised Cochrane risk-of-bias tool for randomized trials (RoB2) [ 40 ] was utilized by two independent reviewers (K.K and S.H) to appraise the selected articles, thus assessing the risk of bias of the included articles. Disputes were settled by discussing with a third reviewer (H. E). Articles with high risk of bias, including studies without a control group and studies in which the randomization was not specified, were excluded from the study. 

### Data Collection Process

A customized form for data extraction was built in Microsoft Excel to classify the details of the studies. These included study ID (first author’s last name and publication date), country of origin, study design, the dosage of the mouthwash and the duration of administration, target population, control group, interval and amount of use, visual analog scale (VAS) score results, salivary flow rate results, sample size, duration of follow up, and risk of bias. 

### Statistical Analysis

The mean difference with standard deviation was calculated for the included studies. Heterogeneity between studies was calculated using the I^2^ and Q indices. In this study, I^2^ value of greater than 50% was considered as significant heterogeneity. For combining the results, a random-effects model was utilized. The Statistical analyses were performed using the CMA v.2.0 software. A probability value less than 0.05 was considered significant. Finally, the results of the meta-analysis were presented in the form of forest plots.

## Results

### Search Results

**Figure 1:**
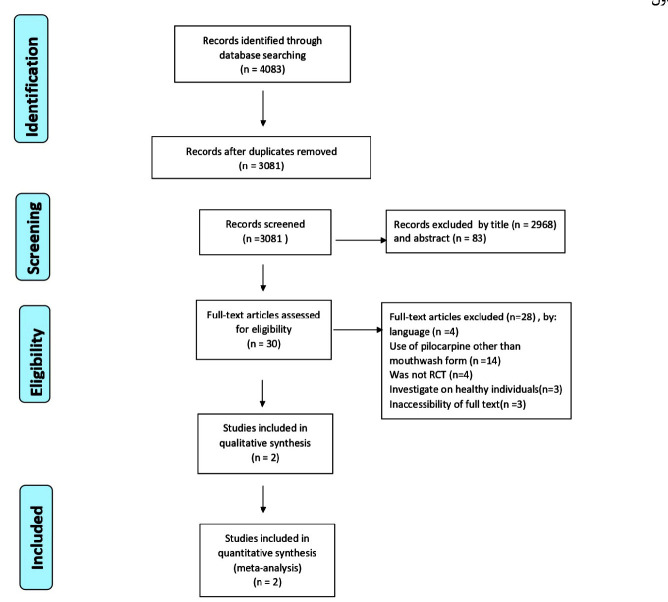
The PRISMA flowchart of the selection process of systematic review

Among 4083 papers initially identified, 3111 studies remained to be assessed after the removal of duplicates. After screening the titles and abstracts, 3080 papers were excluded and the remaining 31 full-text articles were reviewed by two independent reviewers (Figure 1). According to the predefined inclusion and exclusion criteria, three articles were found that examined the effects of pilocarpine in mouthwash form [ 37 - 38 , 41 ]. Although three studies were initially screened, two studies were finally included in the meta-analysis [ 37 - 38 ]. The other study had an open-label and single-arm design [ 41 ].

### The Results of Evaluating the Risk of Bias

**Figure 2:**
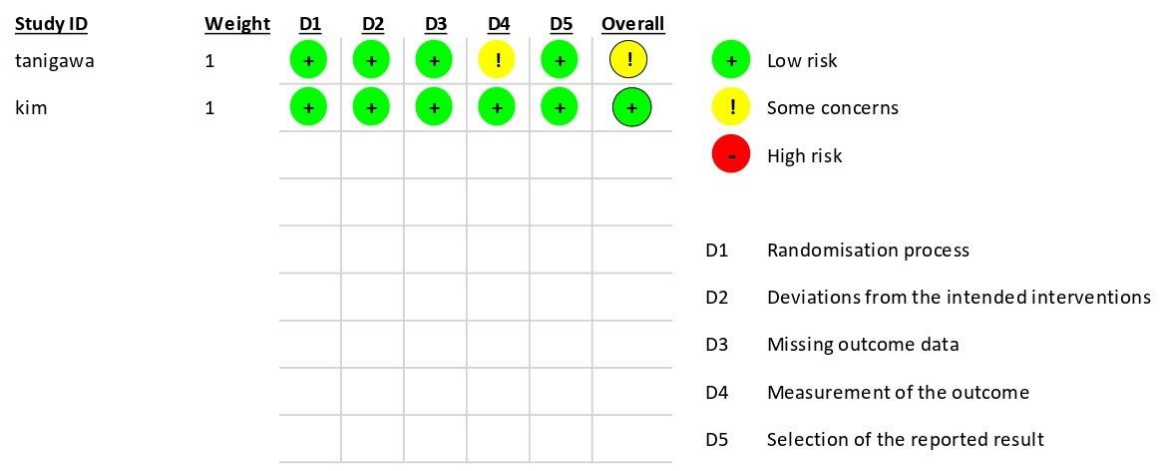
The results of the assessment of the risk of bias

None of the articles showed high risk of bias. According to RoB2, one of the studies showed a low risk of bias [ 37 ], while the other one had a moderate risk of bias [ 38 ]. The details are presented in Figure 2.

### Characteristics of the Studies

The descriptive characteristics and the associated data of the included studies are presented in Table 1. Both papers were prospective randomized double-blind controlled trials. The total number of participants for VAS assessment was 46 in the case (pilocarpine) group and 40 in the control group. Sample size for salivary flow rate evaluation was 43 in the intervention group and 33 in the control group. Mean age of participants was 62.5±3.1 years. Follow-up time varied from 30 minutes to 4 weeks. Both studies used only a single concentration of pilocarpine as an intervention, and no variation in the prescribed pilocarpine concentration was observed in any of the two studies.

**Table 1:** Descriptive characteristics and related data from included studies

Study ID	Country of origin	Sample size (Pilocarpine/ Control) for VAS	Dosage of mouth wash	Duration, interval and amount of use	Initial VAS	VAS After 4 weeks	Initial salivary flow rate (ml/min)	Salivary flow rate (ml/min) after 4 weeks	Risk of bias
J. H. KIM-2014 [ 1 ]	Korea	53 (27/26)	0.1%	1 minute 3times/day 10ml	Case* 5.1±2	4.6±2.5	UWS 0.2±.11	UWS 0.25±.15	Low
					Control 5.7±1.7	5.1±1.6	0.18±.13	0.17±.14	
Tanigawa.T-2015 [ 2 ]	Japan	33 (19/14)	0.01%	2 minutes No defined interval MV: 150 ml/day	Case † 7±1.29	4.79±1.31	SWS 0.71±.14	SWS 0.88±.7	Moderate
					Control 7.07±0.8	6.64±.99	0.83±.12	0.86±.17	

DB RCT = double blind randomized controlled trial, VAS =visual analog scale, MV = maximum volume, UWS= unstimulated whole salivary, SWS= stimulated whole salivary

* =All statistical results of this study are reported on mean ± standard deviation

† = All statistical results of this study are reported on mean ± standard error of measurement

Objective and patient-reported indicators for measuring xerostomia relief were included in the study’s endpoints. The salivary flow rate in mL/min was used as objective data. The changes in Xerostomia symptoms were assessed using a visual analog scale.

### Meta-Analysis

#### Salivary Flow Rate

Due to the divergence of the studies and the different follow-up intervals for this variable, meta-analysis was not possible.

#### VAS Score

Due to the different follow-up intervals, meta-analysis was only possible for VAS results of the follow-up interval of four weeks after using the mouthwash. 

**Figure 3:**
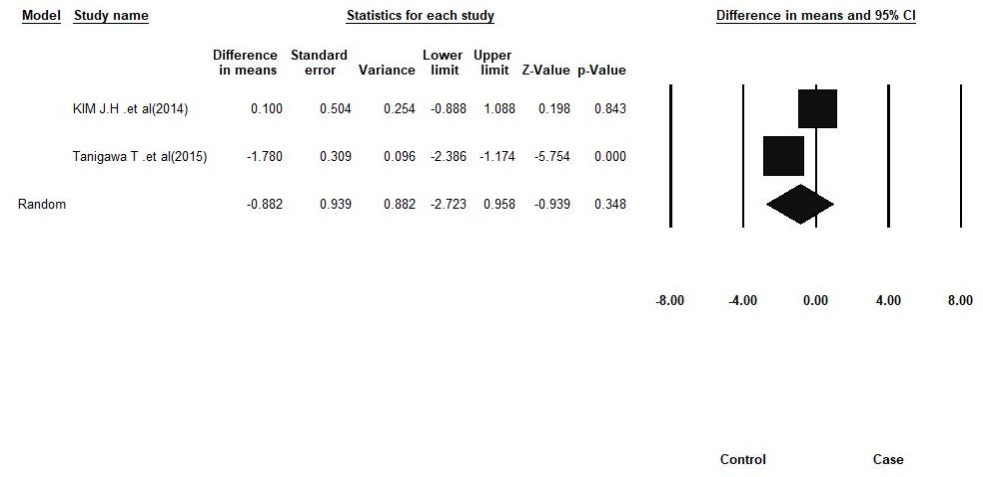
The forest plot of the overall outcome

Heterogeneity of the data was significant (I^2^=90.10, Q-value=10.10, df=1, *p*= 0.001). According to the results of the meta-analysis using the random-effects model, the mean VAS score in people with dry mouth treated with pilocarpine mouthwash was 0.88 units lower than that of the control group four weeks after the intervention. However, it was not statistically significant (Pooled Mean Difference=-0.88, 95% CI= (-2.72; 0.95), *p*= 0.34). Figure 3 shows the forest plot related to the composition of the meta-analysis results.

## Discussion

In the present study, the effects of pilocarpine as mouth-wash on salivary flow rate and the relief of xerostomia symptoms in patients with xerostomia were reviewed. Our systematic review included three comparative studies. We excluded all studies that used topical pilocarpine (such as spray, drop, chewing tablets, candy-like pastille) and had a systemic effect (e.g., swallowing the remaining solution and not spitting it out) to achieve the quite pure topical effect of pilocarpine on dry mouth.

Both unstimulated whole salivary (UWS) and stimulated whole salivary (SWS) flow rates play a role in assessing the rate of dry mouth in patients; however, studying UWS is preferred because it is related to the overall comfort of the patient [ 36 ].

Among the included studies, Kim *et al*. [ 37 ] investigated the effects of 0.1% pilocarpine mouthwash on the UWS of patients with xerostomia, while Tanigawa *et al*. [ 38 ] assessed the effects of 0.01% pilocarpine mouthwash on the SWS of elderly participants with xerostomia.

Kim *et al*. [ 37 ] reported that mouth washing with pilocarpine for four weeks was not significantly different from 0.9% saline in increasing the UWS flow rate. On the other hand, Tanigawa *et al*. [ 38 ] concluded that after using pilocarpine mouthwash for 1 month, the SWS flow rate of case group was significantly higher than the control group.

Despite the results for the long-term effects, the UWS flow rate 60 minutes after mouth washing was significantly higher in the pilocarpine group than in the control group in the study by Kim *et al*. [ 37 ].

In 2018, Watanabe *et al*. [ 41 ] came up with a new combination of mouthwash called pilocarpine/sodium alginate solution and investigated its efficacy and adverse effects in individuals with Sjögren’s syndrome. This article had an open-label and single-arm design, showing that the new formulation improved xerostomia symptoms and the quality of life in patients with Sjögren’s syndrome by increasing the salivary flow rate [ 41 ]. A pilot study in 2021, which studied the effectiveness and safety of local pilocarpine drops for xerostomia in elders, concluded that topical pilocarpine could relieve xerostomia symptoms in patients with ≥70 years of age without significant side effects [ 35 ].

In the present systematic review, papers that examined the healthy individuals were excluded to focus on the main purpose of using pilocarpine, i.e., the treatment of dry mouth in patients. 

Kim et al. [ 37 ] studied the amount of saliva produced by minor salivary glands as key target glands of topical pilocarpine. As a result, palatal and labial secretions differed significantly in short-term evaluation (immediate, 30min, and 60 min after washing the mouth) between the groups but not the buccal secretions.

Because dry mouth is a combination of objective and subjective criteria, and the sensation of dry mouth may be felt even in patients with standard salivary flow rate, studying VAS score is of great importance.

Kim *et al*. [ 37 ] reported that in both short-term (immediate, 30 minutes, and 60 minutes after application) and long-term (4 weeks) effect assessment, despite a significant difference in VAS score results in each of the study groups before and after the treatment, no significant difference was observed between two groups.

Tanigawa *et al*. [ 38 ] reported that the VAS score decreased significantly in case group after four weeks of intervention, and this decrease was not significant in the control group. However, there was no report of differences between the two groups in the study.

While the reason for this inconsistency is unidentified, two probable explanations may involve the frequency of pilocarpine usage, which was more in Tanigawa’s trial than in Kim’s (at any time needed vs. 3 times per day) and the duration of mouth washing with pilocarpine, which was longer in Tanigawa’s trial than in Kim’s (two minutes vs. one minute) [ 37 - 38 ].

In the meta-analysis, the VAS score difference between the two groups after the intervention was not statistically significant.

None of the studies reported serious side effects following mouthwash use; however, in Tanigawa’s study, mouth (13%), tongue (4%) irritation, and mild chest pain (4%) were reported [ 38 ]. Nonetheless, these side effects dissolved by ceasing the mouthwash use. Watanabe *et al*. reported hyperhidrosis (4.2%), hot flushes (12.5%), headache, dizziness, abdominal pain, and rhinitis (4.2% each) [ 41 ]. It should be noted that all these symptoms were mild, and they were much lower than the side effects caused by pilocarpine tablets. The probability of the systemic effects should not be forgotten when prescribing a high concentration of pilocarpine in mouthwash form.

In general, several factors can affect the results of pilocarpine mouthwash treatment. These include secretory reserve capacity of the affected salivary glands, etiology of xerostomia, age, the contact area of the oral mucosa, mouthwash concentration, frequency of use, duration of administration, the volume of mouthwash, the acceptable taste of mouthwash, the saliva collection method, patient cooperation, patients’ medications, and the presence of systemic diseases.

It should be noted that the secretory reserve capacity of salivary glands is more imperative than the etiology of xerostomia in assessing the response to the pilocarpine mouthwash [ 37 ]. Pilocarpine mouthwash is a direct acting cholinergic parasympathomimetic agent, which is able to stimulate salivary secretion from minor salivary glands by diffusing beyond the mucous membrane of the oral cavity and binding to the muscarinic receptor of minor salivary glands [ 42 - 43 ]. 

As a clinical point of current studies, the volume of pilocarpine used each time may affect the oral mucosal absorption more than its usage time. The limitation of this study is that it only evaluated pilocarpine mouthwash and did not assess the other non-systemic forms of this drug.

## Conclusion

Within the limitations of the available evidence, regarding the effects of pilocarpine mouthwash on the salivary flow rate, it seems that using pilocarpine mouthwash can increase the salivary flow rates. However, no optimal dose and application regimen can currently be suggested due to the high heterogeneity of the data. Regarding the relief of the symptoms by pilocarpine mouthwash, the existing evidence does not support its effectiveness. However, given the limited number of the included studies, further well-designed multi-center and comparable regimens and follow-up times in randomized clinical trials are highly warranted.

## Conflict of Interest

The authors declare that they have no conflict of interest.
